# A Tourette Syndrome/ADHD-like Phenotype Results from Postnatal Disruption of CB_1_ and CB_2_ Receptor Signalling

**DOI:** 10.3390/ijms26136052

**Published:** 2025-06-24

**Authors:** Victoria Gorberg, Tamar Harpaz, Emilya Natali Shamir, Orit Diana Karminsky, Ester Fride, Roger G. Pertwee, Iain R. Greig, Peter McCaffery, Sharon Anavi-Goffer

**Affiliations:** 1 Institute of Medical Sciences, University of Aberdeen, Aberdeen AB24 2ZD, UK; victoria.gorberg@abdn.ac.uk (V.G.); rgp@abdn.ac.uk (R.G.P.); i.greig@abdn.ac.uk (I.R.G.); sharon.anavi-goffer@abdn.ac.uk (S.A.-G.); 2 The Mina and Everard Goodman Faculty of Life Sciences, Bar-Ilan University, Ramat-Gan 5290002, Israel; harpaztamar@gmail.com; 3 Department of Psychology, Tel-Aviv University, Tel-Aviv 69978, Israel; emilya.natali@gmail.com; 4 Chaim Sheba Medical Center, Tel-Hashomer, Ramat-Gan 52620, Israel; okarminsky@gmail.com; 5 Departments of Behavioral Sciences and Molecular Biology, Ariel University Center of Samaria, Ariel 44837, Israel

**Keywords:** Tourette syndrome, vocal tic, motor tic, pre-pulse inhibition, elevated-plus maze, ultrasonic vocalisation (USV), CB_1_ receptor, CB_2_ receptor, endocannabinoid system, ∆^9^-tetrahydrocannabinol (∆^9^-THC), rimonabant, nutrition

## Abstract

Cannabinoid receptor 1 (CB_1_) signalling is critical for weight gain and for milk intake in newborn pups. This is important as in humans, low birth weight increases the risk for attention-deficit hyperactivity disorder (ADHD). Moreover, some children with ADHD also have Tourette syndrome (TS). However, it remains unclear if insufficient CB_1_ receptor signalling may promote ADHD/TS-like behaviours. Here, ADHD/TS-like behaviours were studied from postnatal to adulthood by exposing postnatal wild-type CB_1_ and Cannabinoid receptor 2 (CB_2_) knockout mouse pups to SR141716A (rimonabant), a CB_1_ receptor antagonist/inverse agonist. Postnatal disruption of the cannabinoid system by SR141716A induced vocal-like tics and learning deficits in male mice, accompanied by excessive vocalisation, hyperactivity, motor-like tics and/or high-risk behaviour in adults. In CB_1_ knockouts, rearing and risky behaviours increased in females. In CB_2_ knockouts, vocal-like tics did not develop, and males were hyperactive with learning deficits. Importantly, females were hyperactive but showed no vocal-like tics. The appearance of vocal-like tics depends on disrupted CB_1_ receptor signalling and on functional CB_2_ receptors after birth. Inhibition of CB_1_ receptor signalling together with CB_2_ receptor stimulation underlie ADHD/TS-like behaviours in males. This study suggests that the ADHD/TS phenotype may be a single clinical entity resulting from incorrect cannabinoid signalling after birth.

## 1. Introduction

In 90% of children with Tourette syndrome (TS), the vocal and motor tics central to this condition exist in conjunction with another disorder, most frequently (~50%) attention-deficit hyperactivity disorder (ADHD), which in children is mainly characterised by hyperactivity and high-risk behaviour and/or inattention (predominantly inattentive presentation, predominantly hyperactive–impulsive presentation or combined presentation) [1,2,3]. Currently, the diagnosis of TS with ADHD is clinically defined as two separate disorders, each occurring independently.

The risk for long-term ADHD in humans is increased with low body weight at birth [4]. This clinical evidence suggests that signalling systems that regulate body mass may contribute to TS/ADHD characteristics in patients. One potential candidate capable of eliciting a possible combined Tourette syndrome and ADHD phenotype is the cannabinoid system [5]. However, postnatal triggers that can induce a developmental TS/ADHD phenotype have not yet been demonstrated. Postnatal inhibition of cannabinoid CB_1_ receptor activity may provide such a trigger.

In adult patients, SR141716A (Acomplia^®^, rimonabant), a selective cannabinoid CB_1_ receptor antagonist/inverse-agonist, reduces body weight but also produces psychoactive effects, such as increased stress, anxiety and depression [6]. Similarly, selective CB_1_ receptor antagonist/inverse-agonists reduce body weight by inhibiting milk intake in mouse pups [5]; however, comparable effects of SR141716A were found in CB_1_ receptor knockout mice [7,8]. This suggests that SR141716A (20 mg/kg) may directly act on a target other than the CB_1_ receptor, affecting postnatal development [8]. Alternatively, a different mechanism may be involved. Thus, it is possible that in the presence of endogenous tone, once CB_1_ receptors are blocked by SR141716A, endocannabinoids will activate neighbouring receptors and channels, leading to these developmental changes [9,10]. This process may be further enhanced by the production of endocannabinoids, e.g., from dopamine, to overcome CB_1_ receptor blockade and re-stabilise synaptic activity [9]. Such an alternative target for the endocannabinoids, which govern cell fate, neuronal differentiation and axon patterning, could be the cannabinoid CB_2_ receptor [11,12,13,14,15]. In addition, it is possible that both mechanisms co-exist.

The effect of SR141716A on locomotor activity appears to be dependent on age [16,17,18,19]. Indeed, the cannabinoid system plays a role, together with other systems, in controlling locomotor activity, a disruption of which underpins ADHD [20,21]. In adult male Sprague-Dawley rats, SR141716A (10 mg/kg) reduces locomotor activity [16,17] but not grooming behaviour [17]. In contrast, in young adult male Wistar rats and ICR mice (6 weeks old), SR141716A (10 mg/kg) increases locomotor activity [18,19]. On the other hand, in juvenile C57BL/6J male mice, which have been used to investigate its tic effects, SR141716A (10 mg/kg) reduces locomotor activity but not grooming behaviour [20]. When SR141716A is given to pregnant mothers, it induces long-term hyperactivity in their pups [21]. Most of these studies have prompted conclusions that involve the CB_1_ receptor, though non-selective CB_1_/CB_2_ receptor agonists have been used in some of these studies. Thus, it remains unclear if postnatal blockade of the CB_1_ receptors can induce juvenile/adult hyperactivity.

There is also a large body of evidence that patients with ADHD also have learning problems [22,23]. However, this has not been documented in all adults with ADHD [24]. Dysfunction of the sensorimotor gating appears to contribute to both inattention and learning processes [2,3,25,26,27,28]. Across species, functions of the sensorimotor gating system can be indirectly evaluated via the response to an acoustic startle in the pre-pulse inhibition (PPI) test [25,26,27,28]. Previous studies have shown that in adult rats (11-week-old), SR141716A (5 mg/kg) has no effect on the acoustic startle response [29]. Similar results were obtained when juvenile Swiss mice were injected with SR141716A (1, 3 mg/kg) [30] and in CB_1_ receptor knockout (CB_1_^−/−^) mice [31]. In contrast, AM251 (3 mg/kg), an analogue of SR141716A, significantly reduces the acoustic startle response at specific intensities [25]. However, it remains unclear if postnatal blockade of the CB_1_ receptors can induce long-term effects on attention/learning.

Previous findings also suggest that a non-genetic, selective environmental exposure, which alters the signalling of the cannabinoid system, may contribute to the appearance of vocal and motor tics in juvenile rodents [10,32,33,34,35]. In young rodents, SR141716A induces repetitive behaviours, e.g., head twitches or head shakes, which may represent motor tics [10,34,35], for example, exposure of 11–13 day-old rat pups to SR141716A (20 mg/kg) increases, while CP55940, a potent non-selective CB_1_/CB_2_ agonist, decreases ultrasonic vocalisations (USVs), which may represent vocal tics [33].

Collectively, it remains unclear if blockade of the CB_1_ receptors increases tics, hyperactivity and inattention in one model system. Specifically, this study asked if postnatal disruption of the cannabinoid system can (1) induce ADHD/TS-like behaviours in a single model system of cannabinoid system disruption; (2) result in long-term developmental effects; (3) have dose-dependent effects; and (4) induce similar phenotypes in both males and females. This study has attempted to address some of these important questions, because the above studies were conducted in different rodents, age ranges and environments and mainly in juvenile-young/adult male rodents, while ADHD and TS are neurodevelopmental disorders with a childhood onset and prevalence in boys [2].

## 2. Results

### 2.1. Effects of Postnatal Exposure to 5 mg/kg SR141716A on Male Mice

**Figure 1:**
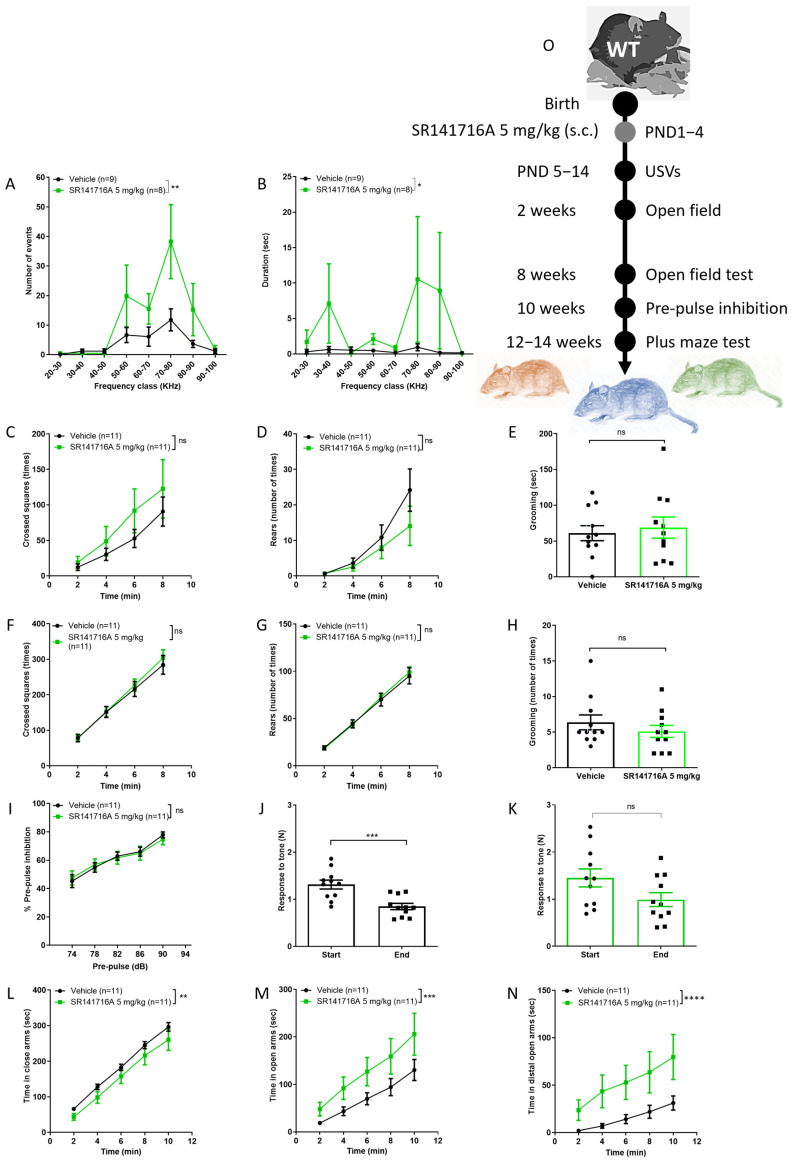
Postnatal exposure to SR141716A 5 mg/kg produced a phenotype of vocal-like tics/ excessive talking and extreme risky behaviour with a reduced learning ability. Mice were tested from age 5 days to 12–14 weeks. The number (**A**) and duration (**B**) of ultrasonic vocalisations (USVs) on postnatal day 14 (PND 14) were significantly increased. The ambulation, rearing and grooming behaviours at age 2 weeks ((**C**–**E**), respectively) and 8 weeks ((**F**–**H**), respectively) were not significantly different between groups. In (**I**–**K**), the effects of SR141716A on the sensory-motor system. SR141716A did not affect the % pre-pulse inhibition to tone at age 10 weeks (**I**). However, the response to tone at the start vs. end of the test was significantly reduced in the ‘Vehicle’ control group (**J**) but not in the ‘SR141716A’ group (**K**). In (**L**–**N**), the effects of SR141716A on anxiety level. The time spent in the closed arm was significantly reduced (**L**), while it was significantly increased in the open arms (**M**) and distal part of the open arms (**N**) in the ‘SR141716A’ group at age 12–14 weeks. (**O**) Experimental scheme. Data represent mean ± SEM. The experiment was independently repeated in 5 different litters. In each graph, *n* represents the number of Sabra males in each group. Line curves were analysed by 2-way ANOVA analysis of variance, followed by Bonferroni’s test for multiple comparisons. Bar graphs were analysed by unpaired, 2-tailed Student’s *t*-test, GraphPad Prism 8 or 9. ns = not significant; * *p* < 0.05; ** *p* < 0.01; *** *p* < 0.001; **** *p* < 0.0001 are significantly different vs. ‘Vehicle’ control group.

Postnatal exposure of male Sabra pups to SR141716A at a dose of 5 mg/kg significantly increased the number and duration of ultrasonic vocalisations (USVs) on PND 14 (Figure 1A,B). In a previous study, acute exposure of 11–13 day-old rat pups to SR141716A increases USVs [33]. Indeed, maternal separation increases USVs in rodents [36], and parental separation increases tics in children [37]. Thus, the increased number of USVs may represent vocal tics, while the increased duration of USVs may represent excessive talking [38]. The ambulation, rearing and grooming behaviours at the age of 2 weeks (Figure 1C–E, respectively) and 8 weeks (Figure 1F,H, respectively) were not significantly different between groups. Postnatal exposure to SR141716A (5 mg/kg) did not affect the % PPI to tone at age 10 weeks (Figure 1I). However, the response to tone at the start vs. end of the test was significantly reduced in the ‘Vehicle’ control group (Figure 1J), while it was not different in the ‘SR141716A’ group (Figure 1H). This suggests that the ‘Vehicle’ control group has developed a memory of the tone and learned it, but the ‘SR141716A’ group has not, suggesting that SR141716A induces memory/learning deficits. Postnatal exposure to SR141716A (5 mg/kg) significantly reduced the time spent in the closed arm at age 12–14 weeks old (Figure 1L), while it significantly increased the time spent in the open arms (Figure 1M) and the distal part of the open arms (Figure 1N), suggesting those in the ‘SR141716A’ group were ready to take more risk. Thus, acute inhibition of CB_1_ receptor signalling by SR141716A (5 mg/kg) after birth resulted in a phenotype of vocal-like tics, excessive vocal communication, intact sensorimotor gate, memory/learning deficits and risky behaviour.

### 2.2. Effects of Postnatal Exposure to 10 mg/kg SR141716A on Male Mice

**Figure 2:**
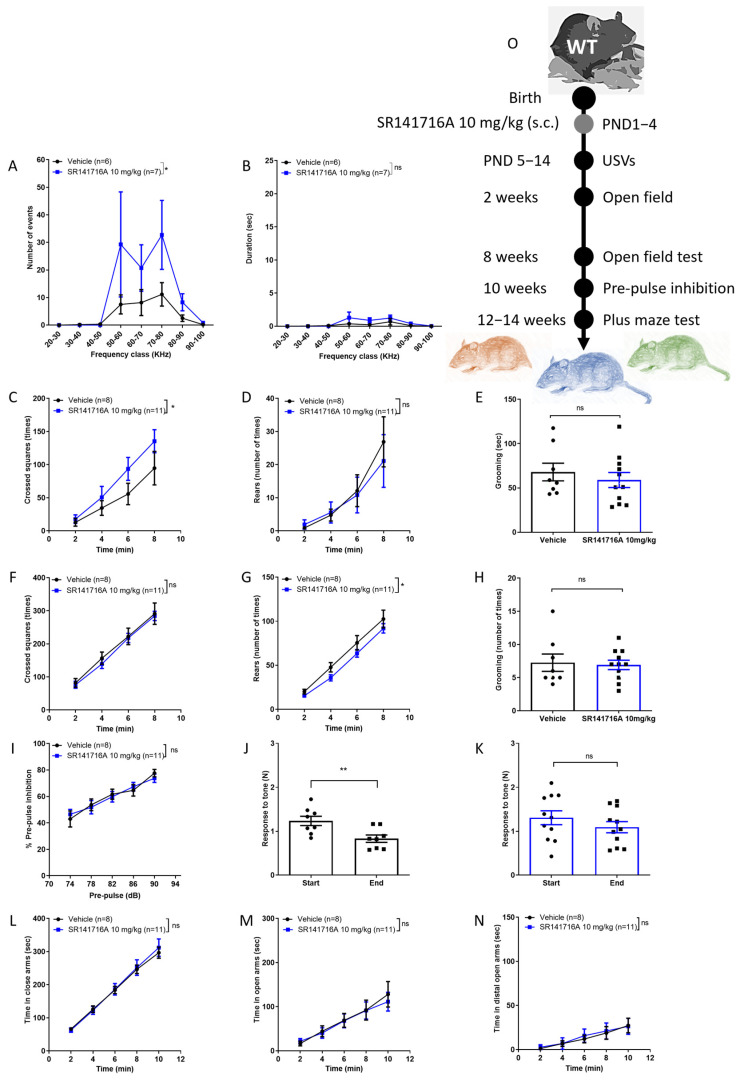
Postnatal exposure to SR141716A 10 mg/kg produced a phenotype of vocal-like tics and hyperactivity at a young age, with reduced learning ability. Mice were tested from age 5 days to 12–14 weeks. At age 14 days, the number (**A**) of USVs was significantly increased but not their duration (**B**). The ambulation, rearing and grooming behaviours at age 2 weeks ((**C**–**E**), respectively) and 8 weeks ((**F**–**H**), respectively) were not significantly different between groups. SR141716A did not affect the % pre-pulse inhibition to tone at age 10 weeks (**I**). However, the response to tone at the start vs. end of the test was significantly reduced in the ‘Vehicle’ control group (**J**) but not in the ‘SR141716A’ group (**K**). At age 12–14 weeks, SR141716A did not affect the time spent in the closed, open or distal part of the open arms ((**L**–**N**), respectively). (**O**) Experimental scheme. Data represent mean ± SEM. The experiment was independently repeated in 5 different litters. In each graph, *n* represents the number of Sabra males in each group. Line curves were analysed by 2-way ANOVA analysis of variance, followed by Bonferroni’s test for multiple comparisons. Bar graphs were analysed by unpaired, 2-tailed Student’s *t*-test, GraphPad Prism 8 or 9. ns = not significant; * *p* < 0.05; ** *p* < 0.01 are significantly different vs. ‘Vehicle’ control group.

In male Sabra pups, postnatal exposure to SR141716A at a dose of 10 mg/kg significantly increased the number (Figure 2A) but not the duration (Figure 2B) of USVs on PND 14. At the age of 2 weeks, the ambulation behaviour was significantly increased but the rearing and grooming behaviours were not (Figure 2C–E, respectively), while at age 8 weeks, the ambulation and grooming behaviours were not different between groups, but the rearing behaviour was reduced in the SR141716A group (Figure 2F–H). Postnatal exposure to SR141716A (10 mg/kg) did not affect the % PPI to tone at the age of 10 weeks (Figure 2I). However, the response to tone at the start vs. end of the test was significantly reduced in the ‘Vehicle’ control group (Figure 2J), while it was not different in the ‘SR141716A’ group (Figure 2H). Postnatal exposure to SR141716A (10 mg/kg) did not affect the time spent in the closed arms (Figure 2L), open arms (Figure 2M) or the distal part of the open arms (Figure 2N). Thus, acute inhibition of CB_1_ receptor signalling by SR141716A (10 mg/kg) after birth resulted in a phenotype of vocal-like tics, normal vocal communication, childhood hyperactivity, intact sensorimotor gate and memory/learning deficits.

### 2.3. Effects of Postnatal Exposure to 20 mg/kg SR141716A on Male Mice

**Figure 3:**
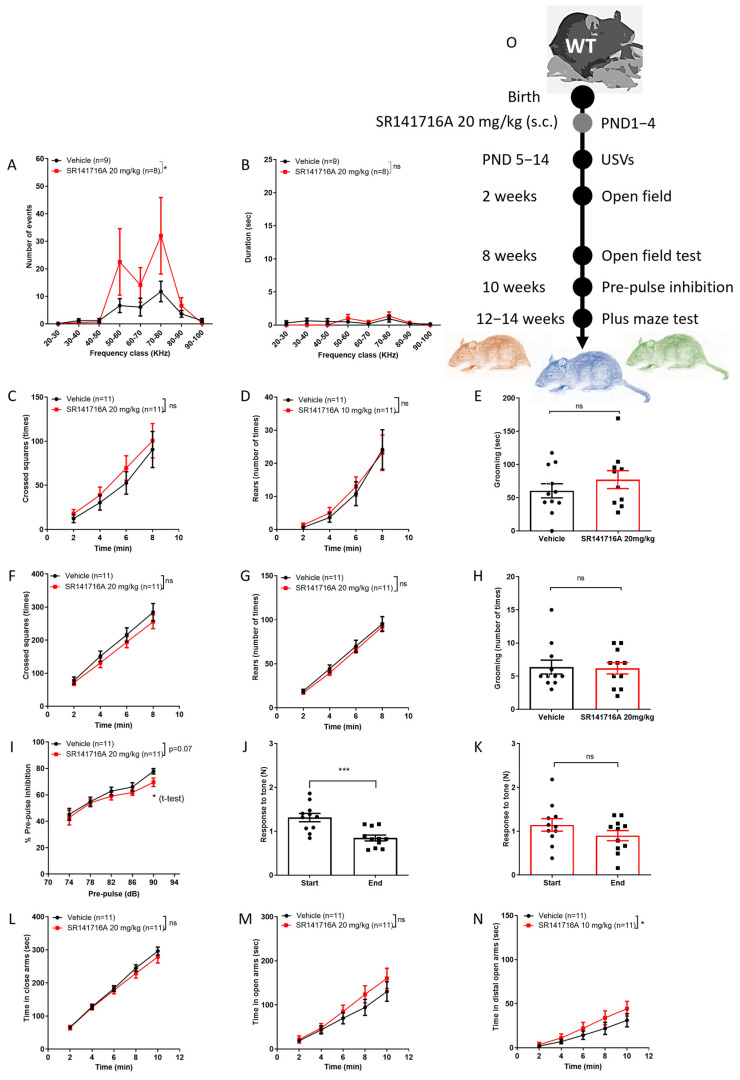
Postnatal exposure to SR141716A 20 mg/kg produced a phenotype of vocal-like tics and some risky behaviour, with reduced learning ability. Mice were tested from age 5 days to 12–14 weeks. At age 14 days, the number of USVs was significantly increased (**A**) but not their duration (**B**). At age 2 weeks, SR141716A significantly increased ambulation (**C**) but not rearing (**D**) and grooming (**E**) behaviours, but not at age 8 weeks ((**F**–**H**), respectively). SR141716A did not affect the % pre-pulse inhibition to tone at age 10 weeks (**I**). However, the response to tone at the start vs. end of the test was significantly reduced in the ‘Vehicle’ control group (**J**) but not in the ‘SR141716A’ group (**K**). At age 12–14 weeks, SR141716A did not affect the time spent in the closed (**L**) and open arms (**M**), but significantly increased the time spent in the distal part of the open arms (**N**). (**O**) Experimental scheme. Data represent mean ± SEM. The experiment was independently repeated in 5 different litters. In each graph, *n* represents the number of Sabra males in each group. Line curves were analysed by 2-way ANOVA analysis of variance, followed by Bonferroni’s test for multiple comparisons. Bar graphs were analysed by unpaired, 2-tailed Student’s *t*-test, GraphPad Prism 8 or 9. ns = not significant; * *p* < 0.05; *** *p* < 0.001 are significantly different vs. ‘Vehicle’ control group.

In male Sabra pups, postnatal exposure to SR141716A at a dose of 20 mg/kg significantly increased the number (Figure 3A) but not the duration (Figure 3B) of USVs on PND 14. The ambulation, rearing and grooming behaviours at age 2 weeks (Figure 3C–E, respectively) and 8 weeks (Figure 3F–H, respectively) were not significantly different between groups. Postnatal exposure to SR141716A (20 mg/kg) affected the % PPI at a pre-pulse of 90 dB at age 10 weeks (* *p* < 0.05 unpaired, two-tailed, Student’s *t*-test; Figure 3I). However, the response to tone at the start vs. end of the test was significantly reduced in the ‘Vehicle’ control group (Figure 3J), while it was not different in the ‘SR141716A’ group (Figure 3H). Postnatal exposure to SR141716A (10 mg/kg) did not affect the time spent in the closed arms (Figure 3L) and open arms (Figure 3M), but it significantly increased the time spent in the distal part of the open arms (Figure 3N). Thus, acute inhibition of CB_1_ receptor signalling by SR141716A (20 mg/kg) after birth resulted in a phenotype of vocal-like tics, normal vocal communication, reduced sensorimotor gate function, memory/learning deficits and risky behaviour.

### 2.4. Effects of Postnatal Exposure to SR141716A on CB_2_ Receptor Knockout Male Mice

**Figure 4:**
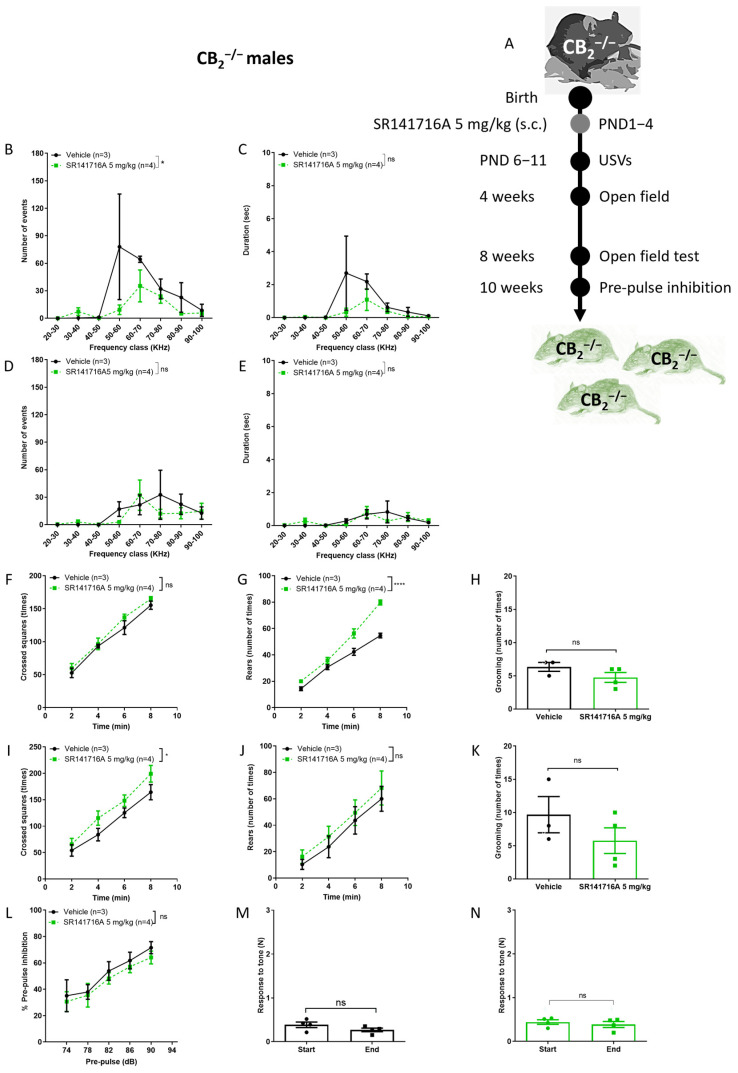
Effect of postnatal exposure to SR141716A 5 mg/kg in CB_2_ receptor knockout mice (CB_2_^−/−)^. (**A**) Experimental scheme. SR141716A significantly reduced the number (**B**) but not the duration (**C**) of ultrasonic vocalisations (USVs) on postnatal day 6 (PND 6), and there was no difference between groups on PND 11 ((**D**,**E**), respectively). The ambulation, rearing and grooming behaviours at age 2 weeks (**F**–**H**) and 8 weeks (**I**–**K**). Rearing (**G**) and ambulation (**I**) behaviours, but not grooming behaviour (**H**,**K**), were significantly increased by SR141716A. In L-N, the effects of SR141716A on the sensory-motor system. SR141716A did not affect the % pre-pulse inhibition to tone (**L**). In CB_2_^−/−^ mice, SR141716A did not affect the response to tone at the start vs. end of the test (**M**,**N**). Data represent mean ± SEM. In each graph, *n* represents the number of Sabra males in each group. Line curves were analysed by 2-way ANOVA analysis of variance, followed by Bonferroni’s test for multiple comparisons. Bar graphs were analysed by unpaired, 2-tailed Student’s *t*-test, GraphPad Prism 8 or 9. ns = not significant; * *p* < 0.05; **** *p* < 0.0001 are significantly different vs. ‘Vehicle’ control group.

In order to determine the on- versus off-target effects of SR141716A [7,39], the effects of SR141716A (5 mg/kg) were tested in CB_2_^−/−^ male mice (Figure 4). SR141716A is a selective antagonist/inverse agonist of the CB_1_ receptor, but at a high concentration may bind to the CB_2_ receptor. In addition, the CB_2_ receptor is relatively highly expressed in the brain at an early postnatal developmental stage [20,40]. Increased numbers of the CB_2_ receptor will reduce the CB_1_/CB_2_ receptor selectivity ratio of SR141716A compared to that in the adult brain. It is possible that activation of the CB_2_ receptor contributes to the reduction of dopamine levels in males [41]. In the absence of CB_2_ receptor expression, SR141716A may only act as a CB_1_ receptor inverse agonist, increasing dopamine release. Thus, we expected that SR141716A would increase locomotor activity and USVs, similar to the effects that were observed in the presence of cocaine [42]. We also expected that if the CB_2_ receptor does not contribute to SR141716A-induced effects on USVs, the vocalisation level of the SR141716A-injected group would overlap with that of the control group.

In male CB_2_^−/−^ mice, postnatal exposure to SR141716A (5 mg/kg) resulted in a significant reduction in the number of USVs on PND 6 (Figure 4A). The duration of USVs was not significantly reduced (Figure 4B). In addition, there was no difference between groups on PND 11; postnatal exposure to SR141716A (5 mg/kg) did not increase the number (Figure 4D) or the duration of USVs in male CB_2_^−/−^ mice (Figure 4E). Importantly, these results provide the first evidence that the development of vocal-like tics is dependent on functional CB_2_ receptors. We expected that if the CB_2_ receptor is not important for SR141716A-induced vocal-like tics, the vocalisation level of the SR141716A-injected group would overlap with that of the control group, but the results show that SR141716A significantly reduced vocalisation below the control group, suggesting that the CB_2_ receptor also has a physiological role in normal vocal communication.

Our results also show that SR141716A (5 mg/kg) significantly increased rearing behaviour at age 4 weeks (Figure 4G) and ambulation behaviour at age 8 weeks (Figure 4I), but grooming behaviour was not significantly reduced (Figure 4H,K). Our results also add to previous findings of the enhanced PPI deficits in CB_2_^−/−^ mice [42]. Postnatal exposure to SR141716A (5 mg/kg) did not significantly reduce the % PPI to tone at age 10 weeks (Figure 4L). However, the response to tone at the start vs. end of the test did not differ between the ‘Vehicle’ control group (Figure 4M) and ‘SR141716’ group (Figure 4N).

Thus, a low level of acute inhibition of CB_1_ receptor signalling after birth of CB_2_^−/−^ mice resulted in a phenotype of reduced vocalisation, hyperactivity and no learning deficits, suggesting that functional CB_2_ receptors are required for normal vocal communication, long-term memory formation and learning processes.

### 2.5. Effects of Diet on SR141716A-Induced Vocal-like Tics in Males

**Figure 5:**
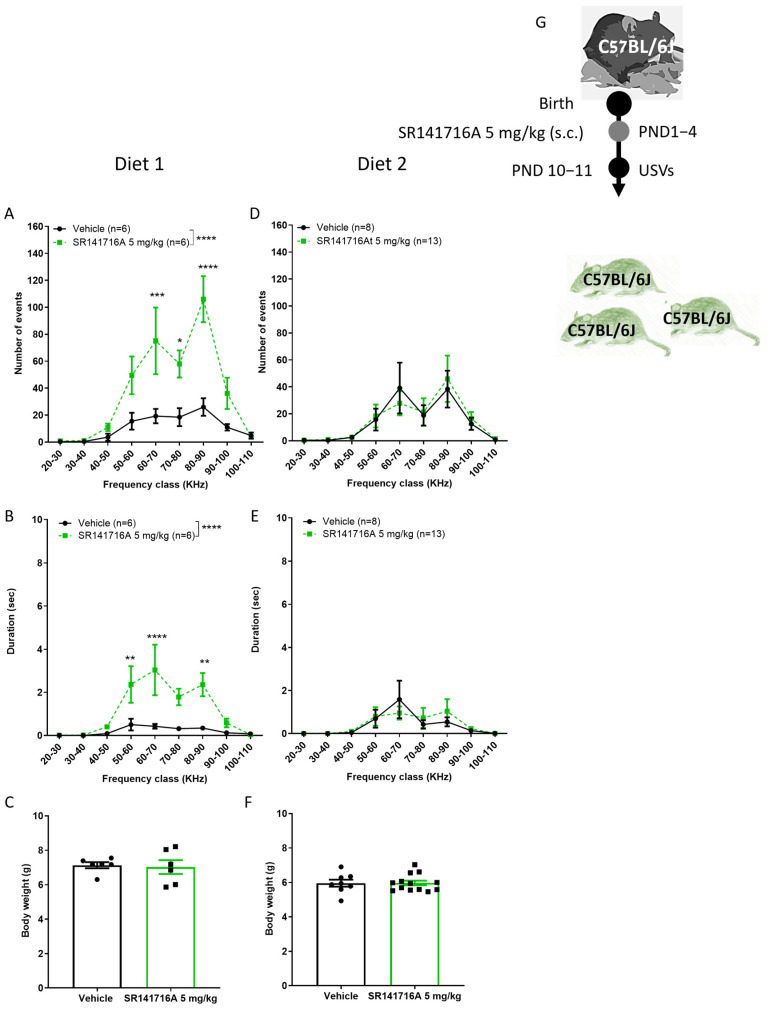
Effect of diets on SR141716A 5 mg/kg-induced ultrasonic vocalisations (USVs). Effect of SR141716A on mice fed with diet type 1 (**A**–**C**) or diet type 2 (**D**–**F**), on the number (**A**,**D**) and duration (**B**,**E**) of USVs. Body weights (**C**,**F**). Experimental scheme (**G**). Each of the parameters was counted in the same mice. Results are expressed as mean ± SEM; *n* represents the number of postnatal day 10–11 male C57BL/6J mice in each group. The experiment was independently repeated in 4 different litters for diet 1 and 6 times for diet type 2. Two-way ANOVA analysis of variance, followed by Bonferroni’s test for multiple comparisons. Bar graphs were analysed by unpaired, 2-tailed Student’s *t*-test. GraphPad Prism 8 or 9. * *p* < 0.05; ** *p* < 0.01; *** *p* < 0.001; **** *p* < 0.0001 are significantly different vs. ‘Vehicle’ control group.

Child and adult mental health has been linked to infant nutritional deficiencies [43,44]. Anecdotal reports suggest that certain foods increase the severity of symptoms in children with Tourette syndrome [45]. However, there is no clear evidence that any diet can reduce the frequency of tics. Here, the hypothesis that a selected optimised nutrient diet controls the incidence of vocal tics in pups was tested using the model of SR141716A-induced vocal tics. Pregnant mice mothers were fed with either diet type 1 or diet type 2, which were optimised for mice, for at least a week before littering. When pregnant C57BL/6J dams were maintained on diet 1, postnatal administration of SR141716A (5 mg/kg) led to an increase in both the number and duration of USVs in C57BL/6J male pups at PND 10–11 (Figure 5A,B), consistent with observations previously described in Sabra male pups at PND 14 (see above). In contrast, when the diet was replaced with diet type 2, there was no difference between the groups; SR141716A (5 mg/kg) did not affect the number or duration of USVs in C57BL/6J male pups nursed by dams maintained on Diet 2 (Figure 5D,E). On the recording day, the body weight of the pups was similar between groups (Figure 5C,F). Thus, the acute disruption in cannabinoid receptor signalling after birth that induces vocal tics is dependent on nutritional nourishment, and an optimised diet can reverse the appearance of vocal tics.

### 2.6. Sex Differences in the Effect of Postnatal SR141716A on Juveniles

**Figure 6:**
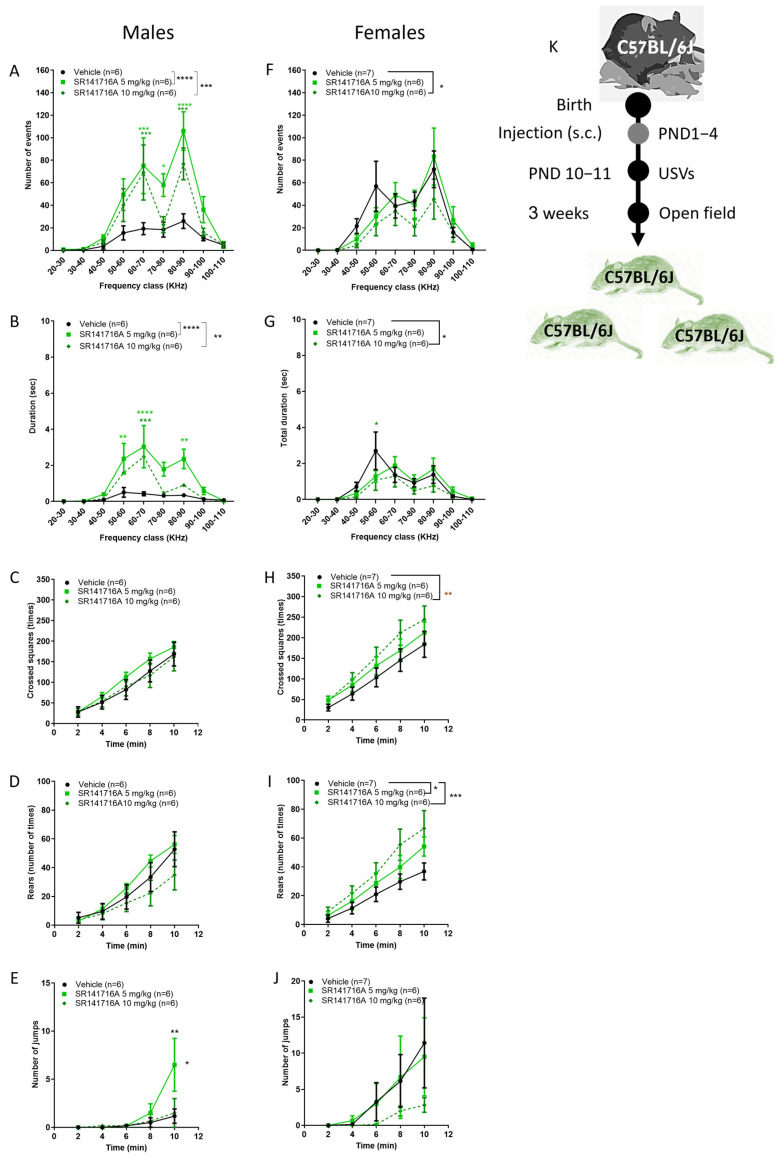
Sex differences in the effects of postnatal SR141716A 5 mg/kg on pups and juvenile mice. Effect of SR141716A on male (**A**–**E**) or female (**F**–**J**) juveniles. Effect of postnatal SR141716A on the number (**A**,**F**) and duration (**B**,**G**) of USVs in pups (PND 10–11) and on ambulation (**C**,**H**), rearing (**D**,**I**) and jumping (**E**,**J**) behaviours of 3-week-old juveniles (PND 18–20). Each of the parameters was counted in the same mice. Results are expressed as mean ± SEM; *n* represents the number of C57BL/6J juveniles in each group. The experiment was independently repeated in 4 different litters (**K**). Two-way ANOVA analysis of variance, followed by Bonferroni’s test for multiple comparisons, GraphPad Prism 8 or 9. * *p* < 0.05; ** *p* < 0.01; *** *p* < 0.001; **** *p* < 0.0001 are significantly different vs. ‘Vehicle’ control group.

The prevalence of Tourette syndrome is about 3 times higher in boys than in girls [2,3]. Therefore, it was relevant to study how SR141716A affects the sexes differently. In C57Bl/6J male pups, postnatal exposure to SR141716A (5, 10 mg/kg) significantly increased the number and duration of USVs in male (Figure 6A,B, respectively) but not in female pups (Figure 6F,G, respectively). In contrast, SR141716A (10 mg/kg) significantly reduced USVs in female pups. In contrast, in the same mice, postnatal exposure to SR141716A (10 mg/kg) significantly increased the ambulation and rearing behaviours in female (Figure 6H,I, respectively) but not in male (Figure 6C,D, respectively) juveniles. In addition, postnatal exposure to SR141716A (5 mg/kg) significantly increased the number of jumps in male (Figure 6E) but not in female (Figure 6J) juveniles. In mice, jumping behaviour is a pattern of escape behaviour that has been proposed as an experimental animal model of panic attacks [46]. In patients with TS, episodes of panic and anxiety increase the number of tics (a tic attack) [47]. As such, jumping behaviour that has been described as motor tics in patients with TS [48] may reflect an archetype of escape behaviour.

Thus, in males, the co-appearance of motor-like tics with vocal-like tics depends on the genetic background. In females, a similar level of acute inhibition of CB_1_ receptor signalling after birth induces hyperactivity but not vocal-like tics. These sex differences suggest that postnatal disruption of cannabinoid receptor signalling contributes to the prevalence of TS in boys.

### 2.7. Contribution of Cannabinoid Receptors to the Effect of SR141716A on Females

**Figure 7:**
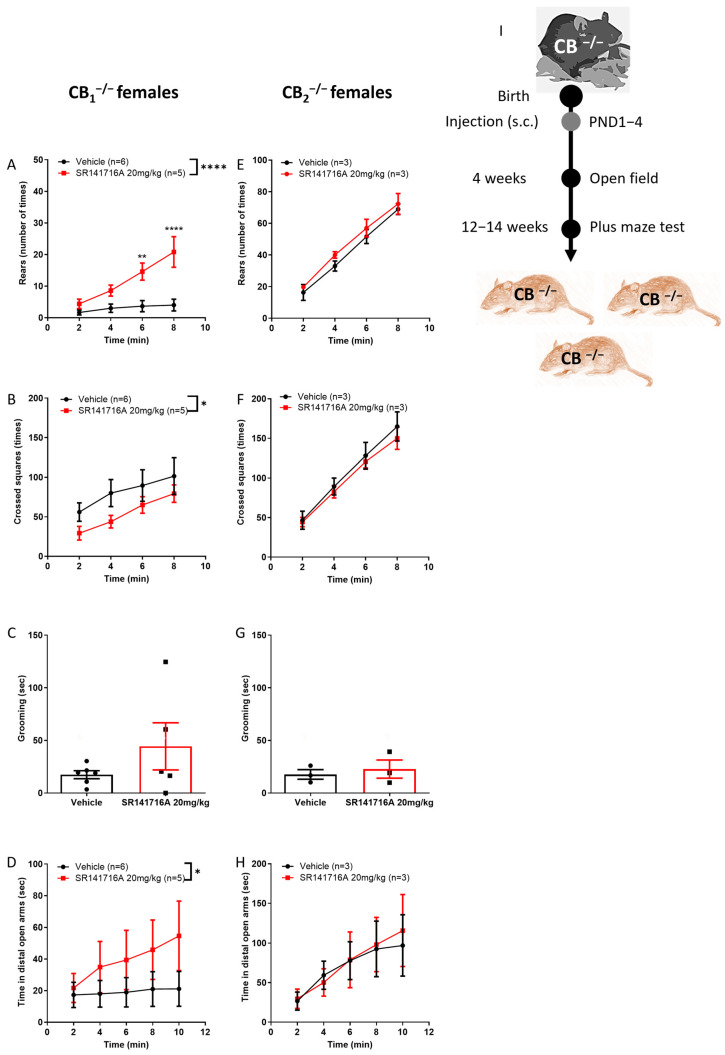
Effect of postnatal SR141716A (SR141716A 20 mg/kg) on female CB_1_^−/−^ and CB_2_^−/−^ knockout mice (**I**). Effect of SR141716A on CB_1_^−/−^ (**A**–**D**) or CB_2_^−/−^ (**E**–**H**) mice. Effect of postnatal SR141716A on rearing (**A**,**E**), ambulation (**B**,**F**) and grooming (**C**,**G**) behaviours of 4-week-old juveniles (PND 23–31), and on the time spent in the distal open arm (**D**,**H**) at age 12–14 weeks Each of the parameters was counted in the same mice. Results are expressed as mean ± SEM; *n* represents the number of mice in each group. The experiment was independently repeated in 3 different litters of CB_1_^−/−^ mice. Two-way ANOVA analysis of variance, followed by Bonferroni’s test for multiple comparisons. Bar graphs were analysed by unpaired, 2-tailed Student’s *t*-test. GraphPad Prism 8 or 9. * *p <* 0.05; ** *p* < 0.01; **** *p <* 0.0001 are significantly different vs. ‘Vehicle’ control group.

The effect of SR141716A on female mice was hypothesised to be entirely mediated by the CB_1_ receptor (Figure 6H,I). Surprisingly, in CB_1_^−/−^ knockout juvenile females, postnatal exposure to SR141716A (20 mg/kg) significantly increased rearing behaviour, while it had no effect on CB_2_^−/−^ knockout juvenile females (Figure 7A,E). Ambulation behaviour was significantly reduced in CB_1_^−/−^ knockout but not in CB_2_^−/−^ knockout juvenile females (Figure 7B,F). Grooming behaviour was not affected in either mouse type (Figure 7C,G). In addition, SR141716A significantly increased the time spent in the distal open arms by the CB_1_^−/−^ knockout but not by CB_2_^−/−^ knockout adult females (Figure 7D,H). Thus, in females, CB_2_ receptor signalling contributes to vertical hyperactivity and risky behaviour, while CB_1_ receptor signalling contributes to horizontal hyperactivity and is required for a normal physiological level of locomotor activity.

## 3. Discussion

This study used an experimental system to investigate the long-term effects of postnatal exposure to SR141716A on TS/ADHD-like phenotypes. USVs in rodents are typically linked to emotional states and environmental stimuli, similar to tics in humans. In this model, USVs may therefore serve as a representation for vocal tics characteristic of Tourette syndrome [33,38]. This interpretation is supported by the following: (1) previous studies have suggested that USVs may serve as a rodent model for vocal tics observed in TS [38]; (2) CB_1_ receptors are densely expressed in brain regions implicated in the pathophysiology of TS, including striosomes and substantia nigra dendron bouquets, circuits that are essential for motor control [49] and are also known to play a role in the postnatal formation of striatal circuits [50]; and (3) aripiprazole, an antipsychotic medication that has demonstrated clinical efficacy in reducing tics in individuals with TS [51], has also been shown to reduce USVs in rodent models [38], suggesting a potential overlap in pharmacological responsiveness.

In line with previous studies, SR141716A was not injected within the first 24 h of life [8] and, thus, did not induce apparent developmental changes nor changes in grooming behaviour [1,7,20]. In male mice, postnatal disruption of the cannabinoid system resulted in a combination of phenotypes, including vocal-like tics with excessive vocalisation, hyperactivity, peripheral motor-like tics (e.g., jumping) in juveniles, and long-term memory or learning deficits and/or risk-taking behaviour in adulthood. In contrast, in female mice, under the same experimental conditions, SR141716A induced a phenotype of hyperactivity without vocal-like or motor-like tics. These results are in line with clinical findings, which documented that low weight at birth is associated with ADHD, which can persist up to the age of 40, though symptoms of ADHD in adults may look different from during childhood [4].

These findings further suggest that acute postnatal disruption of the cannabinoid system after birth contributes to the development of TS/ADHD phenotypes in boys and to the development of hyperactivity in girls. This study suggests that the prevalence of TS in juvenile males depends, at least in part, on the correct signalling of the cannabinoid system after birth.

Emerging evidence suggests that TS and ADHD may share a common neurodevelopmental origin involving dysregulation of the endocannabinoid system [52]. Genetic studies have identified overlapping risk loci and pathway enrichments, particularly within corticostriatal circuits that are essential for motor control and attentional regulation pathways known to be modulated by CB_1_ receptor signalling [53]. Both preclinical and clinical data further support this association; cannabinoids such as Δ^9^-tetrahydrocannabinol have been shown to alleviate tics and hyperactivity in individuals with TS and ADHD [54]. Taken together with the findings of this study, these findings raise the possibility that TS and ADHD may reflect phenotypic variants of a shared neurodevelopmental disorder, potentially initiated by acute postnatal disruption of cannabinoid receptor signalling, specifically involving CB_1_ inhibition and CB_2_ stimulation. Furthermore, supporting these observations, the acute administration of SR141716A to juvenile mice increases forebrain dopamine and serotonin release [55], and conditional deletion of CB_2_ receptors on dopaminergic neurons contributes to the hyperactivity of knockout mice [56]. Indeed, these neurotransmitter systems are the targets for current drug treatment of patients with TS and ADHD [2].

Several repetitive behaviours in rodents, such as ear scratching, excessive grooming, and jumping, have been proposed as models of motor tics, particularly those involving the peripheral nervous system [32,48,57]. Other studies have shown that in juvenile mice (3-week-old) acute injection of SR141716A (1–20 mg/kg i.p.) induces motor-like tics of the head and neck and ear scratch response [20,34,55]. However, while SR141716A increases scratching behaviour in juvenile mice, grooming behaviour is increased in young adults but not in juvenile mice [19,20,34]. Similarly, in young adult Sprague-Dawley rats, SR141716A (2.5 and 5 mg/kg) increases both ‘wet dog shakes’ and ‘head dog shakes’, but it also increases grooming behaviour [10]. In this study, postnatal exposure to SR141716A did not alter grooming behaviour. While the impact of SR141716A on motor tics and premonitory urges in children is unknown, these were not observed in adult humans after taking SR141716A.

In this study, SR141716A-induced vocal-like tics appeared at an earlier age in C57BL/6J mice compared to Sabra mice. This may reflect differences in genetic background, as well as other environmental influences that can affect developmental timing. Moreover, this study found that functional CB_2_ receptors were required for the correct development of vocalisations. This was evident by the (1) shift in the postnatal day the maximal number of vocals appeared; (2) different vocalisation patterns compared to mice expressing the CB_2_ receptor (shown in 2 strains Sabra and C57BL/6J); (3) SR141716A-induced increases in the number of vocalisations (this study and [33]). In addition, this study discovered that functional CB_2_ receptors are required for SR141716A-induced vocal-like tics; and (4) significantly reduced number of vocalisations induced by SR141716A in CB_2_^−/−^ mice. These results suggest that stimulation of the CB_2_ receptor (1) is required for correct development of vocalisations; and (2) induces vocal tics. In males, but not in females, stimulation of CB_2_ receptors may indirectly occur by acute inhibition of CB_1_ receptor signalling after birth. In support of these results, recent studies suggested that under healthy physiological development, stimulation of the CB_2_ receptors reduces the release of dopamine [56,58].

Collectively, disruption of the CB_1_ receptor signalling after birth, together with dysfunctional CB_2_ receptors, may result in a phenotype of ‘quiet’, hyperactive males with memory/learning deficits, which stems from dysfunctional CB_2_ receptors rather than from a CB_1_ receptor blockade. These results add to those obtained in our previous study on the role of CB_2_ receptors in controlling motor tics [20] and further support the hypothesis that disruption of cannabinoid receptor signalling may account for the prevalence of TS/ADHD in boys.

It is well established that a poor diet during child development can lead to poor physical and mental health [43,44]. Indeed, the consumption of caffeine and sugar has been associated with increased frequency of tics, while other parents report that certain allergens in food may exacerbate tic-related symptoms (reviewed by [45]). However, there is still no clear evidence that a specific diet can alleviate tics [45,59,60].

This study found that diet affects the impact of disrupted cannabinoid signalling on the development of TS/ADHD phenotypes. In this model system, diet was found to affect the incidence of SR141716A-induced vocal-like tics in males. Although the study examined the overall effects of dietary type rather than isolating specific components, notable differences were observed in the omega-6 to omega-3 fatty acid ratio and vitamin B6 levels.

Previous reports have indicated that nutritional supplements, including omega-3 fatty acids, were associated with self-reported reductions in both vocal and motor tics in children with TS [61]. Additionally, a pilot study found that omega-3 supplementation combined with Korean Red Ginseng reduced ADHD symptoms in children [62], and certain omega-3 fatty acids have been shown to attenuate seizures in mice through CB_1_ receptor-mediated mechanisms [63]. Furthermore, vitamin B6 has been reported to reduce tic severity and associated anxiety in children with TS in a pilot study [64]. Together, these suggest that diet may play a contributory role in modulating TS-related tics. Importantly, these results suggest that each trigger on its own does not necessarily induce tics, but the combination of altered cannabinoid signalling together with a specific diet may contribute to the appearance of tics in childhood. In line with these results, 11–18-year-old patients with TS reported that a nutritionally enriched diet helped them reduce motor (70% of adolescents) and vocal (50% of adolescents) tics [65]. Thus, early nutritional intervention during child development may help to reduce the incidence of tics. The results of this study further support the importance of nutrients in early brain development [43].

A comparison of the nutritional differences between the two common mouse diets, Teklad Global 2018S (diet 1) and No. 3 breeding (diet 2), suggests that the development of tic-like behaviour is not dependent on a specific component in the diet. These results indicate that there is a need to develop protocols of nutritionally enriched diets, e.g., using lipid-based nutrient supplements, for children with TS. Such supplements are already produced by the World Health Organization (WHO) as complementary foods for infants and young children [66].

## 4. Materials and Methods

### 4.1. Animals

The experimental procedures described below were approved by the Institutional Animal Use and Care Committee of Tel-Aviv University, Ariel University, and the University of Aberdeen in accordance with the UK Home Office, EU directive 63/2010E, and the Animal (Scientific Procedures) Act 1986.

Sabra mice were from Envigo, Israel. C57BL/6J mice were purchased from the Medical Research Facility (University of Aberdeen, UK). CB_1_^−/−^ mice were from Professor Andreas Zimmer (University of Bonn, Germany). CB_1_^−/−^ mice were genotyped with PCR according to the instructions provided by Professor Zimmer [67]. The primers were CB_1_ common: ctc ctg gca cct ctt tct cag tca cg; CB_1_ knockout: tct ctc gtg gga tca ttg ttt ttc tct tga; and CB_1_ wildtype: tgt gtc tcc tgc tgg aac caa cgg. CB_2_ receptor knockout (CB_2_^−/−^) mice were from Jackson Laboratory (JAX #005786; USA). CB_2_^−/−^ mice were genotyped with qPCR according to the instructions provided by the Jackson Laboratory.

The experiments were performed with male and female mice between postnatal day 2 and 14 weeks. Animals were housed in a 12:12 h light–dark cycle at 24 °C, with *ad libitum* access to food and water. For each set of experiments, pups were housed in the same cage with the same mother. In some cases, pups were cross-fostered on postnatal day 2–4 between mothers to ensure sufficient numbers of mice in each group. Cross-fostering and keeping each set of mice with the same mother reduced variation (e.g., in feeding), enabling a reduction in the number of animals. All mice were included in the experiments unless their development was atypical (e.g., eyes did not open, an abnormality in the tail or ears).

### 4.2. Experimental Procedures

Each litter, comprising both male and female pups, was divided into predetermined experimental groups, a control group (vehicle) and a treatment groups (SR141716A 5, 10, 20 mg/kg). Each mouse received a single dose of either SR141716A or vehicle. Within each litter, male pups in the control group served as controls for the male treatment group, and female pups in the control group served as controls for the female treatment group. Pups were randomly assigned to a dose of SR141716A within a litter. These procedures reduced variations between the experiments (e.g., in feeding). Each mouse received a single postnatal injection of either SR141716A (5, 10, or 20 mg/kg) or vehicle and was subsequently assessed across all behavioural tests, from the early postnatal period through to adulthood. The same animals were tested throughout the study, as detailed in the figures below. Body weights were measured on the experimental day or before drug injections. Details related to the number of mice, litters, sex, drug, dose and injections are described below and in figure legends.

Early brain development is impacted by nutritional support [43]. The question was asked whether the varied diets used in different animal facilities may influence the behavioural effects we saw with cannabinoid treatment. We conducted the experiments on C57BL/6J mice maintained on two different, commonly used rodent diets, which were found to influence the postnatal response to SR141716A. The main differences between the diets are detailed in the accompanying references. Diet 1 (Teklad Global 2018S) was sourced from Envigo, UK, and is routinely used in studies involving Sabra mice, which were used to initiate the study. Diet 2 (Rat and Mouse No. 3 Breeding Diet) was obtained from Special Diets Services, Essex, UK, and is the standard diet provided in the C57BL/6J breeding facility. Although the study commenced with the C57BL/6J mice on diet 2, the animals were later switched to diet 1 to replicate the dietary conditions used in previous experiments.

The on-versus off-target effects of postnatal exposure to SR141716A (5 mg/kg), a CB_1_ receptor antagonist/inverse agonist, were tested in CB_2_^−/−^ mice. A model similar to that previously published for assessing the on-versus off-target effects of postnatal HU-308 exposure [20], a CB_2_ receptor–selective agonist, was used [68]. We expected that SR141716A would have similar effects to those observed in the presence of cocaine [42].

Sex differences were studied as mentioned above. The contribution of each cannabinoid receptor to the postnatal exposure to SR141716A (20 mg/kg) was compared between CB_1_^−/−^ and CB_2_^−/−^ knockout female mice.

### 4.3. Drugs and Materials

SR141716A was synthesised by Dr Iain R. Greig, University of Aberdeen, UK (according to US Patent 5,462,960). SR141716A (5, 10, 20 mg/kg) was dissolved in a ‘vehicle solution’ consisting of DMSO, Cremophor EL^®^, and sterile saline in a ratio of 0.6:1:18.4, respectively. DMSO and Cremophor EL^®^ were obtained from Sigma-Aldrich. Control animals received the same ‘vehicle solution’ without the active compound. The drugs were freshly prepared, aliquoted and stored at −20 °C for up to 3 months. SR141716A (or vehicle control) was subcutaneously (s.c.) injected on postnatal day (PND) 1–4 (by weight ~2 g). An aliquot was discarded after one use, and all injections were performed at a volume of 10 µL/g.

### 4.4. USV Recording

It has been suggested that the USVs emitted by juvenile spontaneously hypertensive rats are a representation of TS vocal tics [38]. Notably, postnatal administration of SR141716A (20 mg/kg), a CB_1_ receptor antagonist/inverse agonist, has been shown to increase USVs in rat pups [33], which may represent vocal tics [38]. CB_1_ receptors are densely expressed in striatal striosomes and substantia nigra dendron bouquets, circuits critical for motor control, and have been shown to be implicated in TS pathophysiology [49]. Moreover, CB_1_ receptors are critical for postnatal striatal circuit formation [50]. Aripiprazole, an antipsychotic medication, was shown to effectively treat tics in patients with TS [51], has also been shown to reduce USVs in rodent models [38]. This evidence supports the hypothesis that USVs may serve as a representation for vocal tics associated with TS. Both USVs and tics are linked to emotional states and environmental stimuli in rodents and humans, respectively. Here, we tested the effects of SR141716A on mouse pups. Pups with their mother were habituated in their home cage to the experimental environment for 60 min. In order to evaluate the effect of SR141716A on USVs, both the control group and the experimental groups were tested under the same conditions to control for mother-isolation USVs. Each pup was randomly taken from the home cage, its body weight measured, and the pup was placed in the centre of a polycarbonate cage 21 × 15 × 14 cm, the cage itself was placed inside a polystyrene white box 29 × 23 × 19 cm or 40 × 33 × 24 cm (internal dimensions). The lid of the polystyrene box had two holes for two microphones and holes to allow air inside the box. USVs were electronically recorded on PND 5–14 for 5 min with an UltraSoundGate 416Hb Recorder, Avisoft Bioacoustics, Glienicke/Nordbahn, Germany. The markings of each pup were identified after the recording session, and the pup was then returned to its home cage. The polycarbonate cage was cleaned with 70% ethanol between each pup. USV recordings were made with Avisoft-recorder software version 4.2.27 from Avisoft Bioacoustics, Germany. Background noise was removed. The number and duration of USVs were analysed between 20 and 120 KHz with SASLabPro software version 5.2.13 from Avisoft Bioacoustics, Germany.

### 4.5. Open Field Test

The locomotion test was performed similarly to the methods previously described [68]. Mice were habituated in their home cage to the experimental environment for 60 min. At age 2–3 and 8 weeks, the open field test was performed in a clear glass apparatus 30 × 40 (Sabra mice, CB_1_^−/−^ KO mice, CB_2_^−/−^ KO mice) or 30 × 30 cm (C57BL/6J mice), divided into squares of equal size (black marks). Each mouse was placed in the centre of the apparatus, and movements were manually tracked every 2 min for 8–10 min. Ambulation is the number of times the mouse moves from one square to another with all four limbs. Rearing is the number of times the mouse reared on its hind limbs when front limbs were in the air or placed on the glass, but not if it groomed itself. Grooming is the time in seconds or the number of times of self-cleaning with forelimbs (face, tail, nails or other parts of the body). In patients with TS, jumping behaviour has been described as motor tics [48]. The number of jumps is the number of times each mouse jumped. The body weight of each mouse was documented. The cage was cleaned between each mouse with 70% ethanol. The behaviours were manually counted by pre-trained observers. The observers were blind to the experiments because (1) some tests were conducted by a co-worker who was blinded to the group marking/experiment; (2) some of the tests were conducted days, weeks and even months after injecting the mice; (3) the group marking was randomly assigned; (4) marking was altered between different cages; and (5) the groups were identified after the analysis.

### 4.6. Pre-Pulse Inhibition (PPI) Test of the Startle Reflex

In adults with ADHD, learning deficits are suggested to cause long-term memory deficits [69]. It has been documented that long-term memory in C57BL/6J mice can be predicted from the response to startling stimuli, while the % PPI predicts the performance of the working memory [25]. At the age of 10 weeks, a mouse was placed inside the PPI system (Kinder Scientific, Poway, CA, USA) for 5 min of habituation. Acoustic startles were performed as follows: a pulse was at 120 dB, pre-pulses were at 74–90 dB with 4 dB intervals, each repeated 5 times, and the background tone was at 65 dB. An Excel data sheet with values of jump responses to the acoustic startles (N) was electronically produced. The PPI was calculated from the average values as % PPI = [1 − (startle response for pre-pulse+pulse)/(startle response for pulse alone)] × 100. Responses to the pulse (120 dB) at the start and end of the experiment were compared. Mice were allowed to rest for at least 2 weeks after the PPI test.

### 4.7. Elevated Plus-Maze (EPM) Test

CB_1_ receptors expressed on cortical GABAergic neurones reduce impulsive behaviour-related willingness to take a risk and a comorbid symptom of ADHD [70], while deletion of CB_2_ receptors on dopaminergic neurons increases impulsive and risky behaviour [56]. Thus, based on previous studies [56,70,71,72,73], we assessed within the same EPM test the level of anxiety (more time is spent in the closed arm) and the level of impulsive, risky behaviour (more time is spent in the ‘distal’ part of the open arm, which was the last third of the arm towards the open end) The test was performed when the mice were 12–14 weeks old. Body weight was measured, and each mouse was placed in the centre of the EPM and recorded for 10 min by a video camera (GigE colour ½” Basler acA1300-60gc) and scored using the EthoVision software (version 10, Noldus Information Technology, Wageningen, The Netherlands). Each arm was 5 × 35 cm. The height of the closed arm wall was 15 cm with a 0.5 cm thickness. The experimental room had an upper white light of 3000 K and 600 LM. An Excel data sheet was electronically produced by the EthoVision software, measuring the time spent in each arm. The arms were cleaned between each mouse with 70% ethanol.

### 4.8. Statistical Analysis

All data are expressed as a mean ± SEM. *p* < 0.05 was considered statistically significant. Data were analysed with GraphPad Prism versions 8 or 9 (GraphPad, San Diego, CA, USA). Line curves of USVs, locomotor behaviours, % PPI, and time spent in the arms of the plus-maze were analysed by 2-way analyses of variance (ANOVA), followed by a Bonferroni post hoc test. Post hoc tests were run only if the F ratio was significant, as indicated below (* *p* < 0.05). Bar graphs of body weight, grooming and response to tone in the PPI test were analysed by unpaired, two-tailed, Student’s *t*-test, which was run only if the F-test to compare variance was insignificant. Dose–response results were separated to make it easier to understand the impact of each dose.

## 5. Conclusions

In conclusion, postnatal disruption of the signalling of the cannabinoid system by SR141716A induces a TS/ADHD-like phenotype in young male mice. Functional CB_2_ receptors are required for the development of both correct and tic-like vocalisations. The development of TS/ADHD-like phenotype induced by disrupted cannabinoid signalling is empowered by the nature of nourishment. In female mice, postnatal disruption of the signalling of the cannabinoid system induces hyperactivity. This study suggests that incorrect postnatal signalling of the cannabinoid receptor may contribute to the higher prevalence of TS in boys.

## Data Availability

The data that support the findings of this study are available from the corresponding author upon reasonable request. Some data may not be made available because of privacy or ethical restrictions.

## Abbreviations

The following abbreviations are used in this manuscript:

OCD	Obsessive compulsive disorder
ESR	Ear scratch response
HTR	Head twitch response
∆^9^-THC	∆^9^-tetrahydrocannabinol
TS	Tourette syndrome
ADHD	Attention-deficit hyperactivity disorder
PPI	Pre-pulse inhibition
USV	Ultrasonic vocalization
CB_1_	Cannabinoid receptor 1
CB_2_	Cannabinoid receptor 2
